# Trajectory of external implementation support activities across two states in the United States: A descriptive study

**DOI:** 10.1177/26334895231154285

**Published:** 2023-06-21

**Authors:** William A. Aldridge, Rebecca H. Roppolo, Shannon D. Chaplo, Ariel B. Everett, Sherra N. Lawrence, Christina I. DiSalvo, Devon R. Minch, Jessica J. Reed, Renée I. Boothroyd

**Affiliations:** 1 The Impact Center at Frank Porter Graham Child Development Institute, University of North Carolina, Chapel Hill, NC, USA

**Keywords:** implementation strategies, implementation practice, implementation support practitioner, technical assistance, facilitation, intermediaries, implementation mechanisms

## Abstract

**Background:**

Reporting on strategies to advance implementation outcomes is imperative. The current study reports descriptive information about external implementation support (EIS) provided over 5 years to 13 regions in North Carolina and South Carolina scaling an evidence-based system of parenting and family supports. Regional support teams operating through the Implementation Capacity for Triple P (ICTP) projects employed core practice components (CPCs) for EIS as proposed by Aldridge et al. and further operationalized by members of The Impact Center at FPG Child Development Institute, UNC-Chapel Hill.

**Method:**

Practice activities associated with CPCs were developed and iteratively refined across the study period. ICTP regional support teams systematically tracked their use of CPCs and related activities following each substantive support interaction. Tracking included the duration of time a CPC was employed and the use of specific practice activities associated with that CPC. Data were aggregated by month of the relationship to account for differential start dates across regions.

**Results:**

From November 2016 through December 2021, ICTP support teams tracked 749 support interactions with Triple P regions in North Carolina and South Carolina. Monthly support decreased year over year, though dose varied considerably. Patterns of CPC use indicated a high dose of “foundational” and “co-design” CPCs early, followed by a blended and more diverse use thereafter, with some notable trends. Practice activities considered essential to influencing intended practice outcomes were characterized by higher rates of use. Like CPCs, practice activities were used dynamically across the study period.

**Conclusions:**

This descriptive study offers a case study for how EIS might be operationalized, tracked, and employed. Findings suggest several interpretations that might refine our understanding and use of EIS. Although the nature of this practical report precludes generalizability of findings, directions for future research and practice are discussed.

## Plain Language Summary

More needs to be known about how external support providers work with organizational, system, and community partners to improve their efforts to implement innovative programs and practices. Although a handful of models of external support have emerged in the literature, support strategies or activities are not often tracked systematically or prospectively. In the current study, we report on the adoption, operationalization, and use of a recently proposed model of external support across 13 regions in North Carolina and South Carolina scaling an evidence-based system of parenting and family interventions. Teams of external support providers tracked their activities using the model across more than 5 years. Results provide a description of what external support might look like across long-term support engagements, noting key patterns about dose of support and use of activities that might be responsible for influencing intended support outcomes. The study offers several findings that might refine our understanding and use of external support strategies. In addition, the study lays groundwork for examining additional research questions, such as the feasibility of support processes and whether and how support activities influence support outcomes.

## Introduction

Widespread acknowledgement of inadequate reporting on the strategies used to advance implementation outcomes has led to calls for better monitoring in practice and reporting in the published literature (e.g., Bunger et al., 2017). One cluster of well-recognized implementation strategies identified by Waltz et al., (2015) relates to the provision of interactive assistance, which is generally defined by strategies related to facilitation and technical assistance. At a more detailed level, Leeman et al., (2017) described several capacity-building and scale-up strategies that may be employed by support system actors providing interactive assistance. These include training to build general capacity, technical assistance and facilitation for implementation processes, tools to support implementation processes, quality improvement strategies, infrastructure development, and benchmarking. In this paper, we refer to such strategies and related activities as *external implementation support* (EIS; Aldridge et al., 2023). We refer to providers of EIS as *implementation support practitioners* (ISPs; Albers et al., 2020; Metz et al., 2021). EIS has been recognized for playing a key role in optimizing implementation outcomes (National Academies of Sciences, Engineering, and Medicine [NASEM], 2019) and may be especially useful when scaling multifaceted interventions across communities and complex systems.

Published attempts to systematically and prospectively track strategies related to EIS at an organized level of detail remain rare. In a recent systematic integrative review, Albers et al., (2021) reported on common implementation strategies being used by ISPs as identified through retroactive coding of published reports using the Expert Recommendations for Implementing Change compilation. Their findings suggest that the use of theories, models, or frameworks in the provision of EIS is not consistent and rarely reported with details or theoretically informed explanations of assumed mechanisms of change between support strategies and support outcomes. In fact, the authors reported that no rigorous studies in their review referenced the use of a strategy taxonomy in the design or application of EIS. These findings are similar to those of a synthesis by Katz and Wandersman (2016) in which they described a lack of models, frameworks, or organized approaches related to technical assistance strategies and a lack of quality and rigor in provision of these strategies. The authors concluded that the extent to which technical assistance is being delivered systematically is limited. Although several authors have reported that EIS demonstrates limited effectiveness when considered alone (Albers et al., 2020; Berta et al., 2015; Feinberg et al., 2008; Katz & Wandersman, 2016; McCormack et al., 2013; West et al., 2012), such findings may be in part due to the lack of systematic and theoretically informed delivery of EIS across the field and a lack of rigorous evaluation.

**Figure 1:**
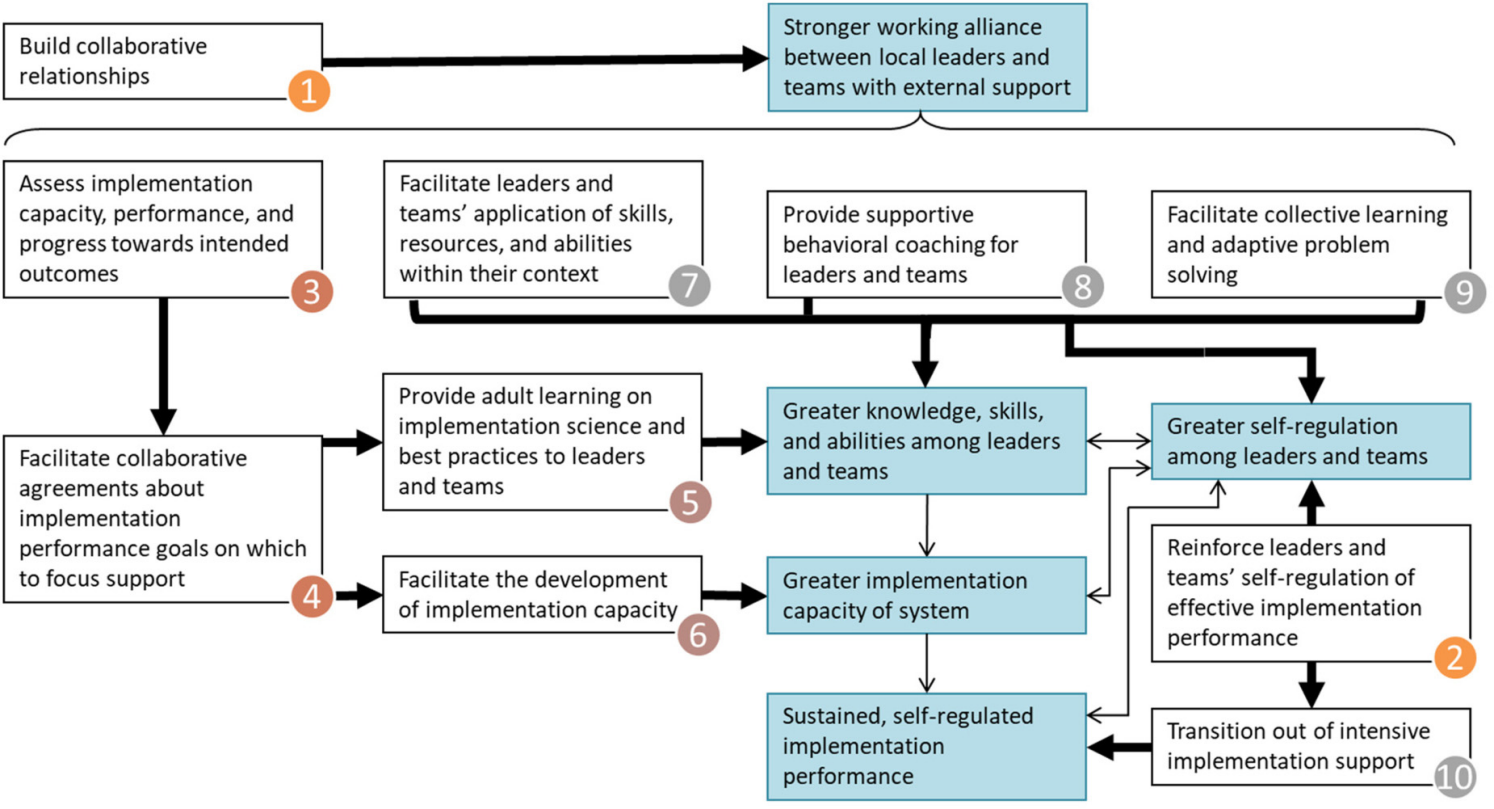
External Implementation Support Theory of Change (Aldridge et al., 2023)

In their effort to better identify and organize strategies that may be central to EIS, Aldridge et al., (2023) proposed 10 core practice components (CPCs), conceptualized as mechanisms of change in EIS, as well as a theory of change for how these practice components may influence immediate-, short-, and long-term EIS outcomes such as implementation capacity and performance (see Figure 1). The model is empirically informed, guided by practice principles, conceptually situated within a grand theory of change for implementation and scale-up (see NASEM, 2019), and grounded in social cognitive theory, which has been widely applied to behavioral change in individuals, groups, organizations, and social systems (e.g., Bandura, 1986, 1988, 1991, 2000; Wood & Bandura, 1989). For more information about this model, including its relation to other EIS concepts and literature, see Aldridge et al., (2023). Early in their development, these CPCs were adopted within The Impact Center at FPG's Implementation Capacity for Triple P (ICTP; https://ictp.fpg.unc.edu) projects. A primary aim of the ICTP projects has been to provide tailored EIS to regions in North Carolina and South Carolina scaling the Triple P – Positive Parenting Program system of interventions. Triple P is an evidence-based system of parenting and family support interventions designed to achieve population-level benefits related to socially important child and family outcomes (e.g., reduction in child maltreatment; Prinz, 2019).

### Operationalizing the CPCs

To ensure that the CPCs were both usable and trackable by ISPs providing EIS to regional partners through the ICTP projects (hereafter referred to as *ICTP regional support teams*), several members of The Impact Center at FPG formed a practice model leadership team to detail *practice activities* that could be used to describe and report on each CPC. As opposed to the more conceptual CPCs, practice activities are the discrete behaviors and activities ISPs may use to influence intended practice outcomes. Three iterations of the operationalization of the CPCs were undertaken across approximately 2 years. The final set of 48 practice activities are reported in Table 1 and have remained unchanged since July 2019. Within this final set, activities are classified as either “essential” or “enhancers.” *Essential activities* (*N* = 28) are hypothesized to directly contribute to the achievement of intended near-term outcomes of each CPC (see Figure 1). *Practice enhancers* (*N* = 20) are hypothesized to accelerate or otherwise enhance the realization of intended near-term practice outcomes, even if outcomes may be sufficiently achievable in their absence. The details of the development and improvement process, including how practices were determined to be essential versus enhancers, are described in Appendix A.

**Table 1:** Core Practice Components and Practice Activities

Core practice component 1	Build collaborative relationships
Activity 1.1^a^	Establish or revise partnership roles, responsibilities, and expectations
Activity 1.2^a^	Provide emotional or practical support regarding hopes, concerns, needs, preferences, and/or context factors
Activity 1.3^a^	Support emotional and practical readiness for next action steps
Activity 1.4	Conduct personal check-ins about work or life more broadly
Activity 1.5	Facilitate the development or revision of group agreements about values and/or behavioral norms for working together
Activity 1.6	During working meeting, provide or share food on- or off-site
Activity 1.7	Engage in social or professional activities outside normal working environment
Core practice component 2	Reinforce leaders’ and teams’ self-regulation of effective implementation performance
Activity 2.1^a^	Reinforce leaders’ and teams’ perceptions of their abilities to apply implementation science/best practices to attain desired performance goals and implementation outcomes (self-efficacy)
Activity 2.2^a^	Reinforce leaders’ and teams’ use of their organizational/system structures, protocols, measures, and tools to manage and improve implementation performance (self-management tools)
Activity 2.3^a^	Reinforce leaders’ and teams’ perceptions of their responsibility for, ownership of, and influence over adaptive organizational change and implementation performance (personal agency)
Activity 2.4^a^	Reinforce leaders’ and teams’ abilities to identify and respond to adaptive and technical challenges to implementation performance (problem solving)
Activity 2.5^a^	Reinforce leaders’ and teams’ perceptions of their abilities to autonomously manage implementation performance, with ongoing support only as needed from external support providers (self-sufficiency)
Core practice component 3	Assess implementation capacity, implementation performance, and progress toward intended outcomes
Activity 3.1^a^	Conduct qualitative interviews (initial or follow-up) to assess implementation capacity and performance within the primary organization receiving support
Activity 3.2^a^	Conduct quantitative assessment of implementation capacity and performance (initial or follow-up) within the primary organization receiving support
Activity 3.3^a^	Assess the primary organization's broader context, current plans, or progress toward desired outcomes, review organizational and/or system records (initial or follow-up)
Activity 3.4	Conduct qualitative interviews (initial or follow-up) to assess implementation capacity and performance *within secondary organizations* being supported by the primary organization
Activity 3.5	Conduct quantitative assessment of implementation capacity and performance (initial or follow-up) *within secondary organizations* being supported by the primary organization
Core practice component 4	Facilitate collaborative agreements about implementation performance goals on which to focus support
Activity 4.1^a^	Facilitate shared understanding about strengths and needs (initial or remaining) related to the implementation capacity and performance of the primary and any secondary organizations
Activity 4.2^a^	Set realistic, jointly conferred goals for establishing or improving *specific* domains of implementation performance within the primary and any secondary organizations
Activity 4.3^a^	Set realistic, jointly conferred strategies focused on the development of organizational resources and abilities in the primary organization to meet each established implementation performance goal
Activity 4.4	Facilitate prioritization of implementation performance domains and goals according to emergent needs, natural sequences, and/or implementation science/best practices
Activity 4.5	Determine multiple “early wins”: Initial action steps that can easily be accomplished to build positive momentum toward the achievement of strategies and implementation performance goals

Core practice component 5	Provide adult learning on implementation science and best practices to leaders and teams
Activity 5.1^a^	Set and monitor (empirically or observationally) the progress of learning objectives related to implementation science/best practices of established implementation performance goals
Activity 5.2^a^	Use independent and/or in-person adult learning strategies to facilitate presentation and practice of implementation science/best practice knowledge and skills related to established implementation performance goals
Activity 5.3	Use incidental or “just in time” learning to strengthen connections between implementation science/best practices and real-time discussions or activities
Activity 5.4	Facilitate the use of online learning resources
Activity 5.5	Broker external learning resources or events
Core practice component 6	Facilitate the development of implementation capacity
Activity 6.1^a^	Working together with leaders and teams, develop, adapt, and/or monitor progress of shared action plans to advance previously identified strategies focused on the development of organizational resources and abilities to meet established implementation performance goals
Activity 6.2^a^	Building on prior implementation science/best practice knowledge and skill learning, facilitate the development or refinement of organizational/system structures, resources, or practices for leaders’ and teams’ management of implementation performance in accordance with established implementation performance goals
Activity 6.3	Link relevant items from capacity assessments (CCA/IDA/IOCA) to shared action plans
Activity 6.4	Utilize PDSA cycles as appropriate to systematically improve the development of organizational/system structures, resources, or practices
Activity 6.5	Broker external partnerships or resources to advance the development of organizational/system structures, resources, or practices
Core practice component 7	Facilitate leaders’ and teams’ application of skills, resources, and abilities within their context
Activity 7.1^a^	Facilitate appropriate, naturally occurring, or intentionally created experiential learning activities related to established implementation performance goals, so that leaders and teams apply knowledge, skills, and organizational/system structures, resources, or practices (whether old or new)
Activity 7.2^a^	Ensure that leaders and teams engage in experiential learning activities within the full context of their system environment
Activity 7.3	Before engagement in experiential learning activities, use anticipatory guidance to promote leaders’ and teams’ success, ensure their safety, and facilitate their anticipatory problem solving related to perceived or potential threats/challenges
Core practice component 8	Provide supportive behavioral coaching to leaders and teams
Activity 8.1^a^	Observe or debrief leaders’ and team members’ experiential learning activities related to established implementation performance goals
Activity 8.2^a^	Build confidence of leaders and team members to achieve established implementation performance goals by providing specific behavioral praise
Activity 8.3^a^	Engage leaders and team members in ongoing independent and/or in-person adult learning activities that promote skill and knowledge refinement for the next application
Activity 8.4	Normalize or temper thoughts and/or feelings related to the application of knowledge or skills (old or new) within local context
Activity 8.5	Promote the generalization of leaders’ and team members’ specific skills and professional judgements to varied applied contexts

Core practice component 9	Facilitate collective learning and adaptive problem solving
Activity 9.1^a^	Facilitate leaders’ and teams’ use of data, feedback, and implementation science/best practices to identify and analyze root causes of barriers, challenges, and lessons learned as they actively pursue established implementation performance goals
Activity 9.2^a^	Hold leaders and teams accountable to what has been agreed upon (including references to prior agreements and expectations)
Activity 9.3^a^	Facilitate leaders’ and teams’ identification of technical and adaptive elements of challenges and the use of appropriate strategies to address them
Activity 9.4	Facilitate leaders’ and teams’ documentation of collective learning and problem solving
Activity 9.5	Facilitate leaders’ and teams’ engagement of key partners for shared learning and help with problem solving
Core practice component 10	Transition out of intensive implementation support
Activity 10.1^a^	Establish a shared rationale for transitioning out of the intensive implementation support
Activity 10.2^a^	Facilitate leaders’ and teams’ understanding of when and to whom they might need to reach out for support in the future
Activity 10.3	Facilitate leaders’ and teams’ identification of the support processes that best contribute to their ongoing success and sustainability

*Note*. CCA = Community Capacity Assessment; IDA = Implementation Drivers Assessment; IOCA = Intermediary Organization Capacity Assessment; PDSA = Plan-Do-Study-Act.

^a^ Essential activities.

### Intended Use of CPCs and Practice Activities

As described by Aldridge et al., (2023), the 10 CPCs used in this study offer multiple pathways to the achievement of intended practice outcomes, target behavior change at both individual/team and organizational/system levels, and are typically dynamic in their use, despite some thematic groupings and sequential dependencies in conceptualization. The final set of practice activities reported in Table 1 build on these characteristics. Support interactions may draw from combinations of individual practice activities across CPCs. That is, ISPs may proactively or responsively combine individual activities across CPCs to tailor support interactions, respond to specific context cues or needs, and influence more than one practice outcome at a time. This approach recognizes the often-dynamic nature of implementation practice, the high level of flexibility and adaptation needed within any given support interaction, and places a premium on ISP experience, judgement, intuition, and skill. Likewise, training and coaching for ISPs and the application of this model in accordance with its guiding theory and practice principles become particularly important (Aldridge et al., 2023). This is not unlike the demands that *intervention practitioners* often face when delivering evidence-based interventions with flexibility while maintaining fidelity to core intervention components and alignment with underlying principles and theories of change (Kendall & Frank, 2018). In our operationalization of the 10 CPCs, fidelity might be understood as ongoing attention to the proposed 28 essential activities and alignment with the underlying principles and theories of change across the support engagement.

Additional details and examples of the intended use of CPCs and related practice activities are provided in Appendix B. Overall, we believe that the 28 essential activities and 20 practice enhancers in Table 1, organized within the 10 CPCs for EIS proposed by Aldridge et al. (2023), offer a way to operationalize ISP actions more thoroughly. They also have the benefits of offering utility and flexibility for ISPs in learning, application, and coaching; being trackable at the level of discrete practice activities rather than categorical support events or strategies; and being situated within a theory of change that connects CPCs to intended practice outcomes. Moreover, we believe this approach helps to conceptualize broader implementation strategies related to EIS in a more usable way for both practice and research.

### Tracking CPCs and Practice Activities in the ICTP Projects

The first *empirical* step in our development of this more fully operationalized practice model has been to track and monitor the use of the 10 CPCs and related practice activities by ICTP regional support teams. As there were three iterations of development and improvement in our operationalization of the CPCs across 2 years (see Appendix A), tracking and monitoring activities included different iterations of the practice activities over time. The original list of 59 practice activities was used to retroactively track EIS within the ICTP projects from November 2016 through July 2017 and prospectively until the start of January 2018. The set of 60 practice activities resulting from the first revision process was then used to prospectively track EIS from early January 2018 through June 2019. The current set of 48 practice activities has been used to prospectively track EIS from July 2019 to date. The purpose of this descriptive study is to catalog and describe the trajectory of EIS CPCs and practice activities within the ICTP projects. We were particularly interested in examining (a) the monthly trajectory of CPC dose provided by ICTP regional support teams and (b) ICTP regional support teams’ use of practice activities across the 10 CPCs.

## Methods

### ICTP Regional Support Teams

ICTP regional support teams are responsible for providing tailored EIS to regions in North Carolina and South Carolina scaling the Triple P system of interventions. Regional support teams have typically consisted of two ISPs partnering with regional Triple P leaders and implementation team members to co-design and facilitate the support process using the 10 CPCs and the various iterations of related practice activities as described in Appendix A and Table 1. Additional details, including training and coaching activities provided to ICTP ISPs, are provided in Appendix C.

#### North Carolina

In North Carolina, the state is divided into 10 regions for Triple P scale-up. With funding from a regional endowment and the state division of public health, ICTP regional support teams started providing tailored EIS to Triple P regions in November 2016, well after statewide scale-up activities were underway, with a demonstration of the support process in one region. This region, inclusive of just one county, was selected through a request for interest and assessment process related to engagement in intensive support, which entailed regular and ongoing support (typically up to 12 onsite and 24 distance support contacts, plus additional distance contacts as needed) with a focus on strengthening broad-based community capacity for scaling the Triple P system. With additional funding from the state division of social services, a second region that had gone through the same request for interest and assessment process was added in February 2017. Starting in October 2018 by request of the state Triple P leadership team, including existing funders, additional pairs of regions were added to the support process every 3–5 months through a similar request for interest and assessment process. To ensure adequate capacity for intensive support as new regions were onboarded to the support process, ICTP ISPs partnered with parenting program specialists from a second statewide intermediary organization to provide tailored EIS from July 2019 through June 2020. By October 2019, all 10 regions were onboarded into intensive support and a variety of universal supports (e.g., online learning modules, facilitated sessions at quarterly statewide learning collaboratives). In July 2020, the ICTP project team resumed sole responsibility for providing broad-based EIS to Triple P regions across the state.

Moreover, in July 2020, ICTP regional support teams added a middle tier of support, having experienced that intensive support was less appropriate for some regions due to (a) the strength of community capacity development for Triple P scale-up in that region, often among regions that had participated in ICTP support or prior implementation evaluation activities, or (b) time-limited contextual challenges, needs, or preferences that precluded full participation in the intensive support process. The middle tier of support was designed to be briefer (typically up to six onsite and 12 distance support contacts, plus additional distance contacts as needed) and more narrowly focused on discrete areas of community capacity for Triple P system scale-up (e.g., leadership and implementation teams, workforce development systems, quality and outcome monitoring systems).

#### South Carolina

In South Carolina, three counties are hosting a Triple P scale-up initiative. With funding from a regional endowment, ICTP ISPs were paired with community capacity coaches (Strompolis et al., 2020) from a South Carolina intermediary organization to initially provide tailored EIS to two start-up counties in October 2018 and January 2019. The South Carolina intermediary organization funds the Triple P backbone organizations in these counties. Backbone organizations were selected using a request for interest and assessment process inclusive of elements related to both Triple P system scale-up and participation in a support process like the intensive support process used in North Carolina. A third county that was added to the support process in May 2019 was funded separately by a local foundation. As they had already built a large amount of community capacity for scaling Triple P and had limited ability to participate in the support process due to having only one Triple P coordinator, this county was provided support more in line with the brief support model used in North Carolina. Support in the initial two counties was fully transitioned to the South Carolina intermediary organization and data collection ceased in July 2020. Support in the third county continued to be provided by partnership between the South Carolina intermediary and the ICTP project team through June 2021, after which support was fully transitioned to the South Carolina intermediary organization and data collection ceased.

Additional information about statewide Triple P scaling contexts and the support partnerships within the present study is available in Appendix C.

### ICTP Support Tracking

To track CPCs and practice activities, we developed an online Qualtrics tracking form to capture details of support contacts. A single member of each regional support team (always the ICTP ISP if the team included an ISP from a partner organization) was asked to report the names of the ISPs, the date and format of the support contact, and the goals of the support contact. To estimate dosage, the duration of time for any CPC employed during the support contact was reported. For each CPC reported, a checklist was completed to record the specific practice activities that were used within that CPC. This tracker was completed following every substantive support contact, including email communication if the regional support team judged that time was spent in support of regional performance goals. ICTP regional support team members were encouraged to complete tracking forms for any support provided by the following week after the support contact. Additionally, regional support team members received weekly calendar reminders and monthly summary emails with current contact logs for the month to prompt completion of forms for any missing support contacts. Contact logs were reviewed monthly by ICTP evaluation team members. Duplicates or errors were flagged for quality control and reviewed by the regional support team member who submitted the form.

### Data Aggregation Across Regions Participating in Support

To better observe how CPCs and practice activities were used over the course of support relationships, data were aggregated by month of the relationship to account for differential start dates across regions; the start dates of support relationships ranged from November 2016 to December 2019. As a result, support data are available from different numbers of regions across time. For example, South Carolina regions transitioned out of ICTP support activities and related tracking on the timelines discussed above, contemporaneously ending their contributions to the data sets used in the current study. Conversely, some North Carolina regions have not yet experienced longer-term support relationships, and thus, support data are not yet available for later years.

### Alignment of Practice Activities Across Iteratively Developed Sets

As three iterative sets of practice activities were used for tracking EIS during the period under examination, there were implications for how descriptive analyses could be carried out. The changes between the first and second iterations of the practice activities were relatively minor overall and allowed for retroactive alignment. However, the final set of practice activities in Table 1 incorporated several significant changes to the number and nature of practice activities, including the identification of *essential activities* and *practice enhancers*. While some retrospective alignment of practice activities to prior iterations was possible, there was not an exact match for all activities. The implication is that analyses related to practice activities are limited to tracking forms that were completed from July 2019 onward.

## Results

From November 21, 2016 through December 31, 2021, ICTP regional support teams tracked 749 support events across the 10 North Carolina Triple P regions and three Triple P South Carolina counties in this study (*n* = 13).

### Overall Dose of EIS

 Table 2 provides a summary of regional support, including month of first support event, months of support provided, average number of support events per month, and descriptive statistics for monthly dose of support provided. Counties in South Carolina tended to have lower average monthly dose compared to North Carolina regions (2.39 h and 3.77 h per month, respectively). Regions that initiated support before 2019 tended to have higher average monthly dose compared to regions that started later (4.20 h and 2.98 h per month, respectively).

**Table 2:** Summary of Support for North Carolina (NC) and South Carolina (SC) Triple P Regions in this Study

Region	First formal support event	Months of support	Average support events per month	Average dose of support per month (hr)	Median (hr)	*SD* (hr)
NC 1	Nov 2016	62	1.95	5.15	4.00	4.71
NC 2	Feb 2017	59	2.41	5.15	3.00	5.27
SC 1	Sept 2018	22	2.68	1.27	1.00	1.22
NC 3	Nov 2018	38	1.74	5.94	3.25	5.93
NC 4	Dec 2018	37	1.70	3.56	1.75	4.34
SC 2	Jan 2019	20	2.15	2.61	2.33	1.38
NC 5	Feb 2019	35	1.37	3.45	2.50	3.37
NC 6	Feb 2019	35	1.60	6.79	6.00	4.72
NC 7	July 2019	30	1.13	2.55	1.50	3.29
SC 3	Aug 2019	23	0.96	3.30	1.83	3.37
NC 8	Aug 2019	29	0.79	2.04	1.00	2.55
NC 9	Nov 2019	26	0.85	1.58	1.46	1.62
NC 10	Dec 2019	25	2.00	1.48	0.75	2.11

As seen in Table 3, EIS generally begins with a high dose of support during the initiation of the support relationship and tapers over time, leveling off at 2–3 h of support per month by the end of Year 1. The greatest average volumes of support per month are observed before 6 months. Monthly support during the 1st year was higher than Year 2, which, in turn, was higher than Year 3 (5.26, 2.71, and 2.57 h, respectively). A different pattern of dosage emerges in later months: a lower baseline with discrete bumps in Months 31–43 and increasing dosage after Month 43, though both trends are reflective of support in a smaller number of regions. Support varies considerably. For example, in 12% of months, regions were provided no support and 10% of support months have over 10 h of support reported.

**Table 3:** Summary of External Implementation Support Dose by Month of Support

Month of support	Number of regions	Average (hr)	Median (hr)	*SD* (hr)	Minimum (min)	Maximum (min)
1	13	8.77	8.00	5.73	45	1,050
2	13	3.69	2.75	3.73	0	820
3	13	9.12	7.00	7.44	50	1,410
4	13	2.17	1.50	1.57	20	320
5	13	8.95	9.92	5.78	0	1,020
6	13	3.42	1.57	3.96	0	780
7	13	4.36	3.50	4.09	0	805
8	13	5.69	4.50	5.22	0	960
9	13	4.10	1.33	4.48	25	870
10	13	3.71	4.58	2.81	0	560
11	13	3.31	1.67	3.46	0	690
12	13	5.77	3.92	6.12	15	1,315
13	13	2.56	1.67	4.11	0	920
14	13	2.22	1.75	2.50	0	480
15	13	2.59	1.17	4.04	0	930
16	13	3.20	1.25	5.43	0	1,050
17	13	3.76	2.25	4.13	0	855
18	13	4.35	2.58	5.39	0	990
19	13	2.47	2.50	1.67	0	270
20	13	2.59	2.67	2.20	0	455
21	12	2.65	1.50	3.91	0	880
22	12	1.96	1.33	1.67	0	380
23	11	1.95	1.00	3.21	0	670
24	10	2.21	1.50	1.82	0	380
25	10	3.61	2.00	4.69	45	1,000
26	9	2.79	2.50	2.06	0	365
27	8	2.75	2.29	2.35	0	385
28	8	2.53	2.71	1.46	40	300
29	8	3.58	1.50	5.63	0	990
30	7	3.77	2.25	4.74	0	845
31	6	1.89	1.92	1.76	0	270
32	6	1.50	1.25	1.67	0	270
33	6	2.11	1.25	2.70	0	450
34	6	2.32	2.00	1.97	0	345
35	6	1.29	1.13	1.47	0	240
36	4	2.73	1.75	3.29	0	445
37	4	1.31	0.63	1.89	0	240
38	3	4.20	5.20	3.60	10	435
39	2	1.00	1.00	1.41	0	120
40	2	2.50	2.50	2.12	60	240
41	2	4.21	4.21	0.65	225	280
42	2	1.00	1.00	0.00	60	60
43	2	0.96	0.96	0.06	55	60
44	2	1.42	1.42	1.30	30	140
45	2	2.38	2.38	1.24	90	195
46	2	2.04	2.04	0.77	90	155
47	2	4.08	4.08	4.01	75	415
48	2	2.04	2.04	0.29	110	135
49	2	2.46	2.46	0.77	115	180
50	2	2.54	2.54	2.18	60	245
51	2	2.00	2.00	1.41	60	180
52	2	3.04	3.04	1.36	125	240
53	2	3.29	3.29	0.29	185	210
54	2	2.00	2.00	0.71	90	150
55	2	4.50	4.50	2.83	150	390
56	2	4.00	4.00	0.71	210	270
57	2	2.50	2.50	1.41	90	210
58	2	6.08	6.08	5.77	120	610
59	2	1.50	1.50	0.71	60	120
60	1	5.00	5.00			
61	1	6.00	6.00			
62	1	1.00	1.00			

### Use of CPCs

**Figure 2:**
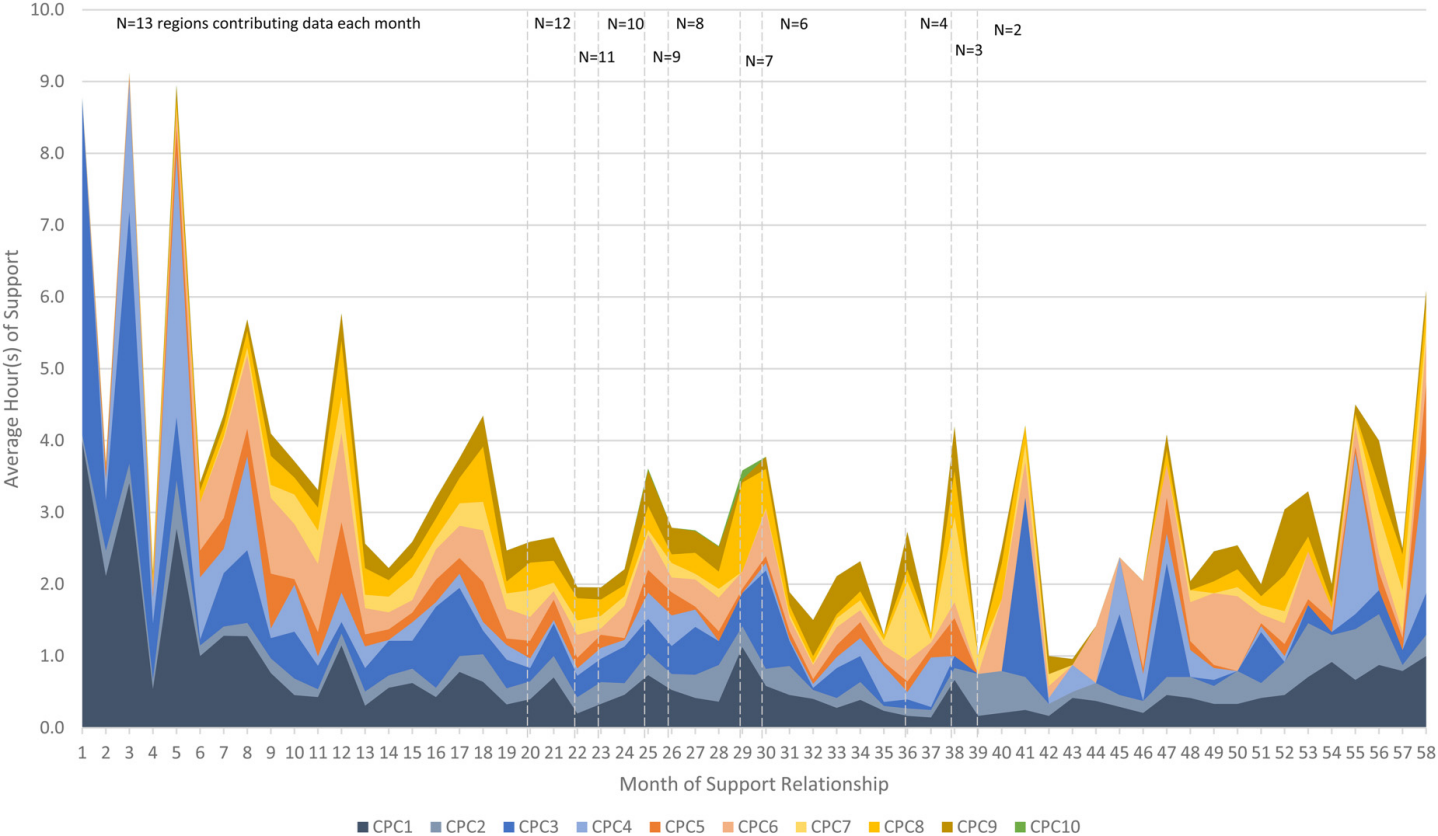
Average Dose of Core Practice Component by Month of Support

Average dose of EIS by CPC was examined to explore the trajectory of their use over time. Figure 2 is a stacked-area chart depicting average CPC dose each month of the support relationship. Not every CPC was used in each support interaction. Table 4 provides descriptive statistics for dose of CPCs in support events in which they were used.

**Table 4:** Average Dose of Core Practice Components during Support Events in Which They Were Used

CPC	Support events using CPC (%)	Average dose when used (min)	*SD* (min)
CPC 1	91 (*n* = 459)	19.9	23.0
CPC 2	65 (*n* = 338)	14.3	10.2
CPC 3	37 (*n* = 189)	50.7	68.4
CPC 4	26 (*n* = 130)	50.6	70.2
CPC 5	31 (*n* = 156)	28.2	32.8
CPC 6	42 (*n* = 212)	30.2	35.9
CPC 7	25 (*n* = 125)	25.1	26.9
CPC 8	42 (*n* = 213)	18.6	16.9
CPC 9	36 (*n* = 183)	22.1	25.8
CPC 10	1 (*n* = 3)	8.3	5.8

Note. CPC = core practice component.

Examining Figure 2 and Table 4, several observations are noteworthy. First, EIS generally begins with a high dose of support comprised mostly of CPCs 1–4, transitioning to a diverse composition of CPCs around the 6-month mark of the support relationship and lasting several years. Second, building collaborative relationships (CPC 1) is the most frequently used core component. Reinforcing leaders’ and teams’ self-regulation of effective implementation performance (CPC 2) is also used consistently across support months, although at a lower dosage than CPC 1. Third, providing adult learning on implementation science and best practices (CPC 5) increases sharply at Month 5 and is frequently employed for the next several months before slowly tapering. Facilitating the development of implementation capacity (CPC 6) follows a similar pattern to CPC 5, though also appears in high doses at later stages of support among the two regions in longer-term support engagements. Fourth, facilitating leaders’ and teams’ application of skills, resources, and abilities within their context (CPC 7) is rarely used until at least Month 7 of the support relationship, one of the latest CPCs to be employed. Average monthly dose of this CPC increases from Months 6–12, declining slowly over the following years of the relationship, with occasional spikes in dose occurring in later years. Fifth, providing supportive behavioral coaching to leaders and teams (CPC 8) is used earlier and at a slightly higher dose than other CPCs that are intended to support regional implementation performance (CPCs 7 and 9), with peaks in dosage at Months 12, 18, and 30. Finally, transition out of intensive implementation support (CPC 10) was used by only three regions during the data collection period: in Months 5 and 7 of the support relationship with region SC 2 for 20 min total; in Month 29 of the relationship with region NC 1 for 60 min; and in Months 25, 27, and 28 of the relationship with region NC 8 for 25 min total.

### Use of Practice Activities

**Figure 3:**
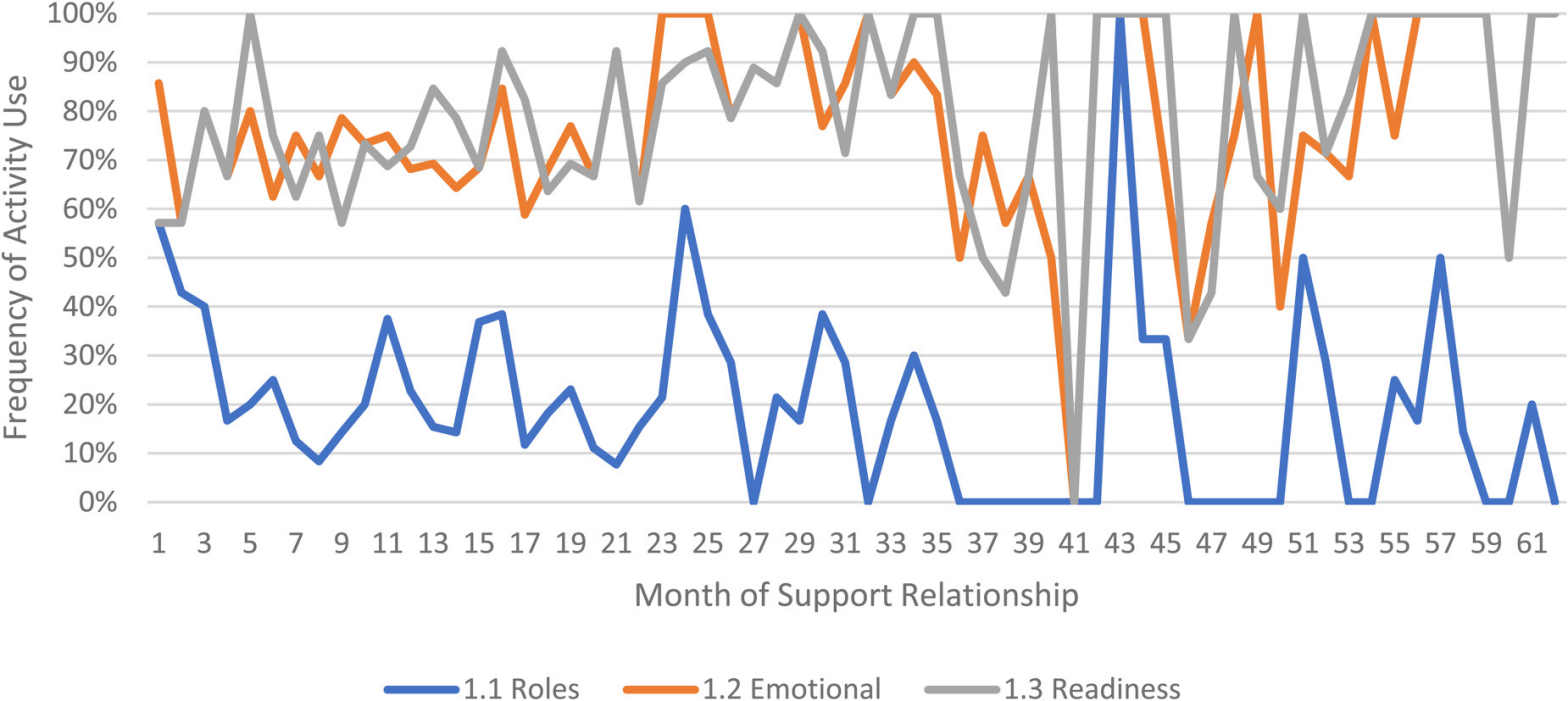
Use of Essential Activities Within CPC 1 (Build Collaborative Relationships) by Month of Support

*Note*. CPC = core practice component.

**Figure 4:**
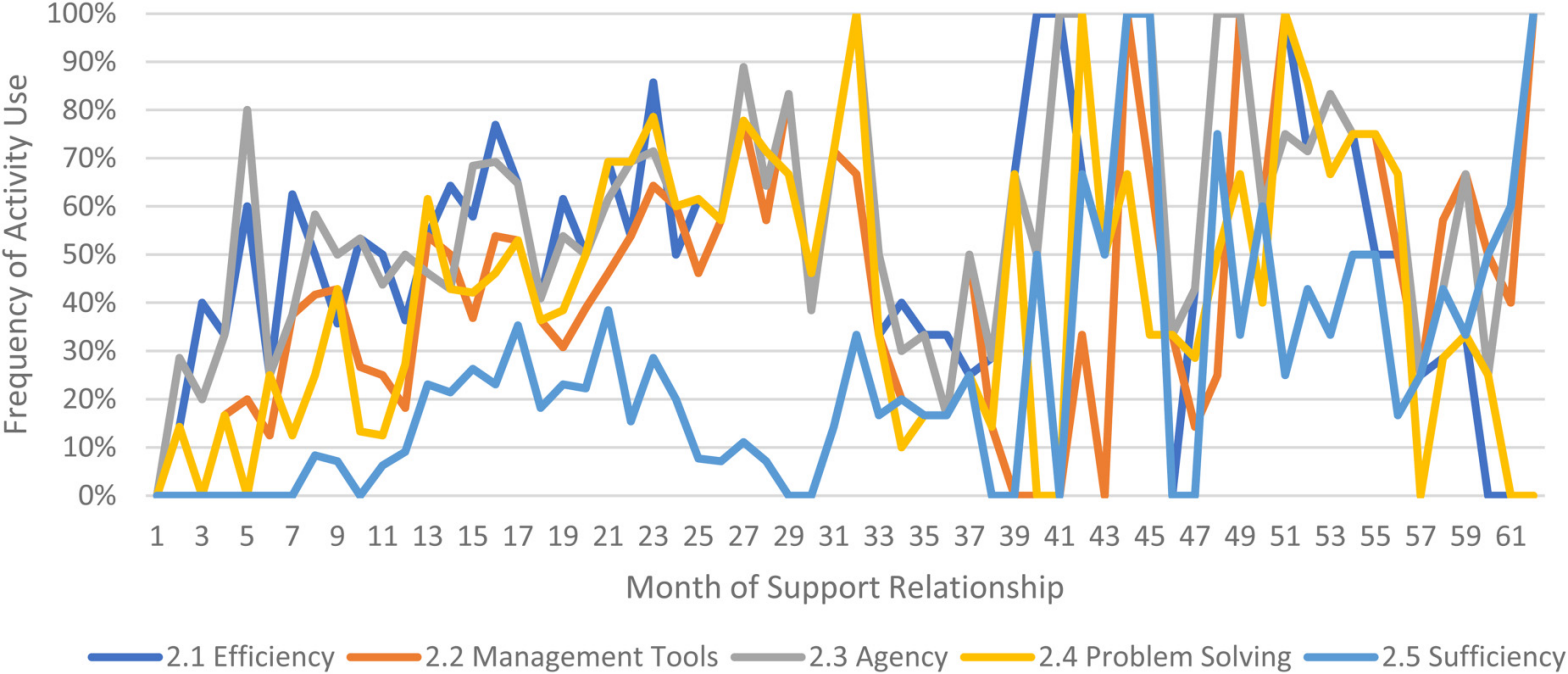
Use of Essential Activities Within CPC 2 (Reinforce Leaders’ and Teams’ Self-Regulation of Effective Implementation Performance) by Month of Support

*Note*. CPC = core practice component.

**Figure 5:**
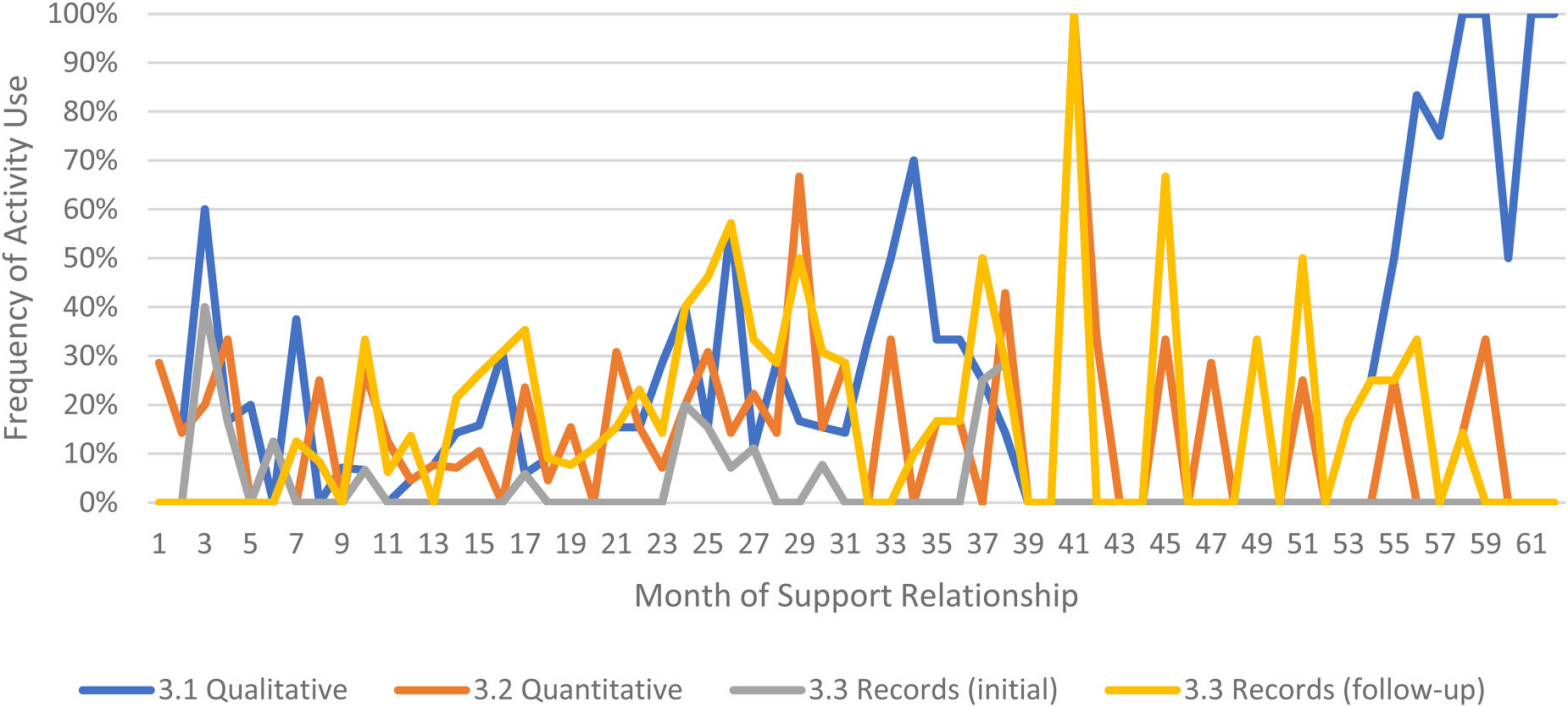
Use of Essential Activities Within CPC 3 (Assess Implementation Capacity, Implementation Performance, And Progress Towards Intended Outcomes) by Month of Support

*Note*. CPC = core practice component.

**Figure 6:**
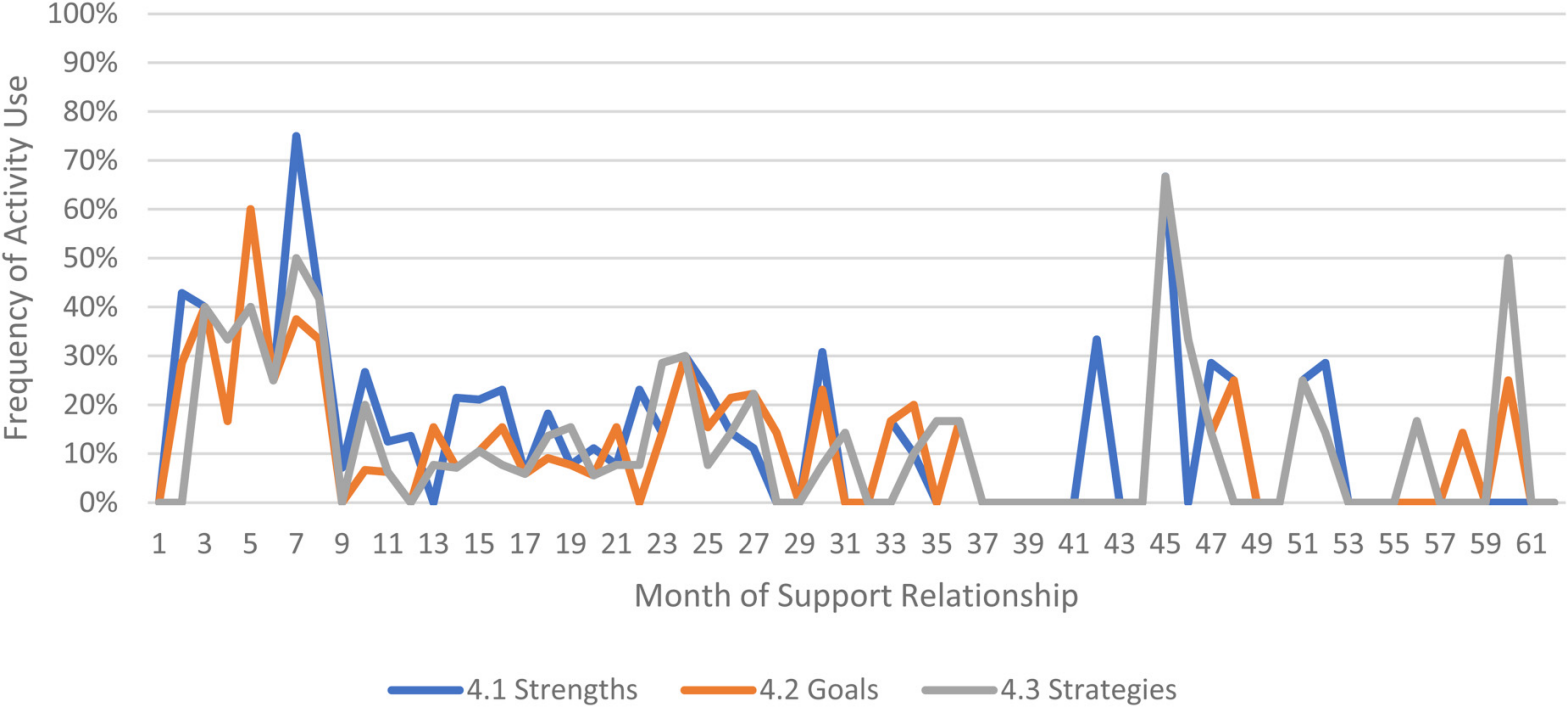
Use of Essential Activities Within CPC 4 (Facilitate Collaborative Agreements About Implementation Performance Goals on Which to Focus Support) by Month of Support

*Note*. CPC = core practice component.

**Figure 7:**
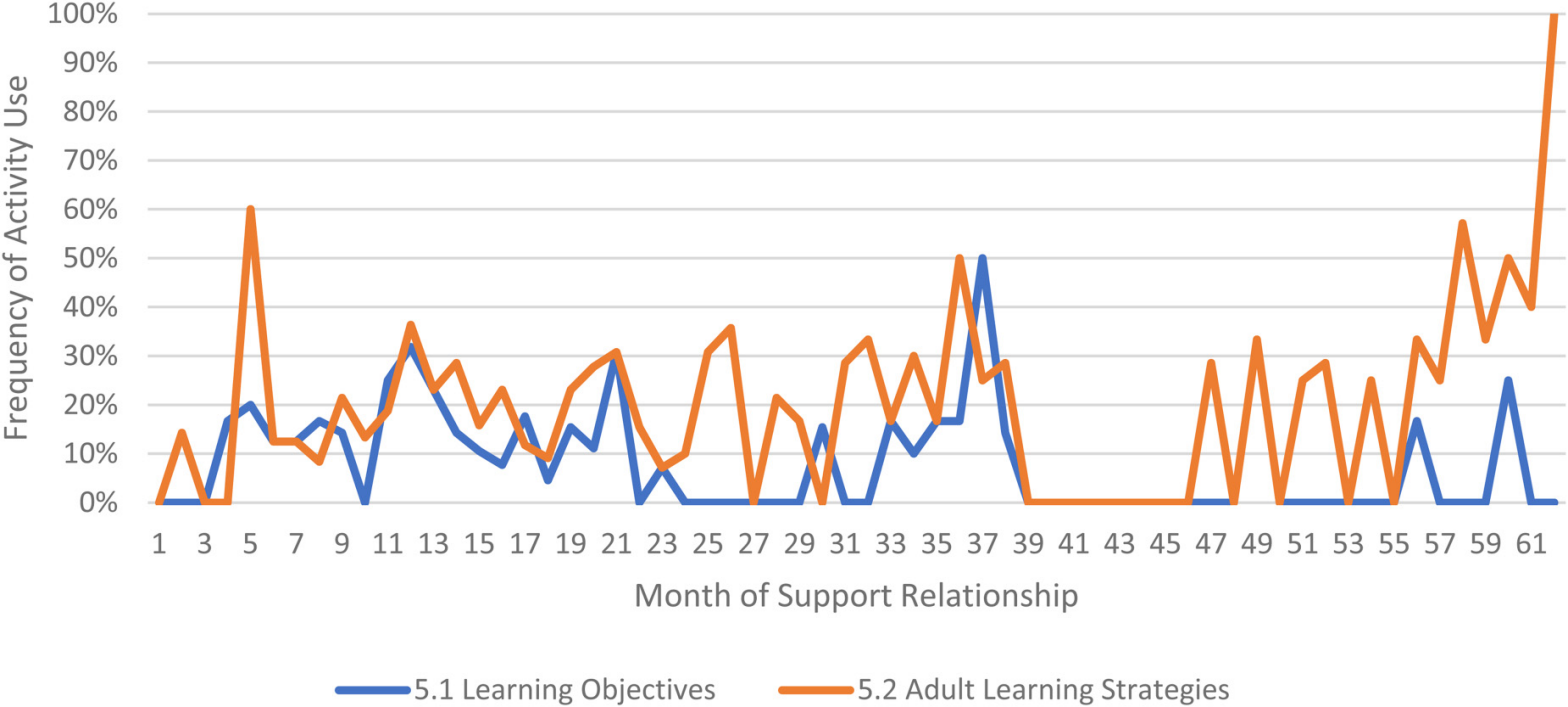
Use of Essential Activities Within CPC 5 (Provide Adult Learning on Implementation Science and Best Practices to Leaders and Teams) by Month of Support

*Note*. CPC = core practice component.

**Figure 8:**
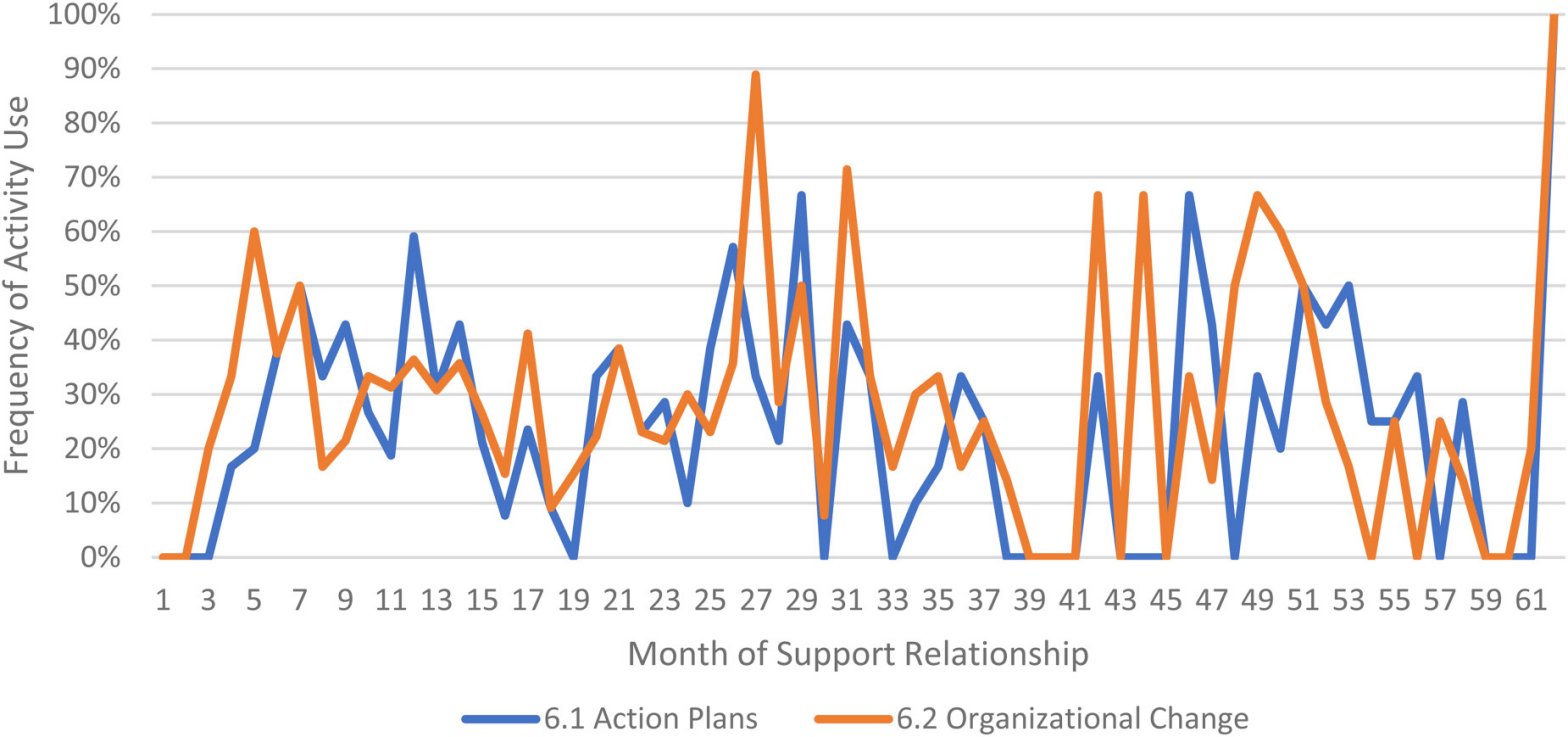
Use of Essential Activities Within CPC 6 (Facilitate the Development of Implementation Capacity) by Month of Support

*Note*. CPC = core practice component.

**Figure 9:**
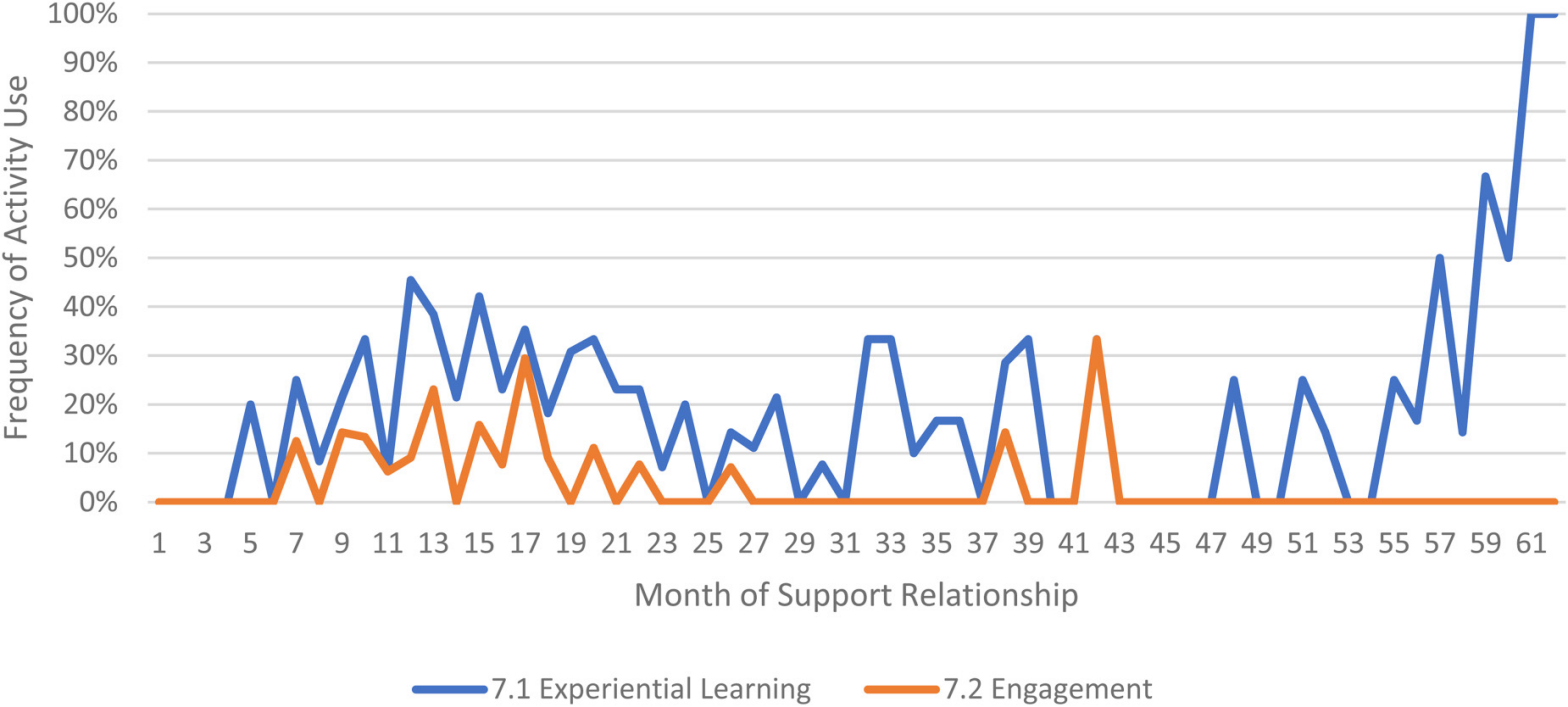
Use of Essential Activities Within CPC 7 (Facilitate Leaders’ and Teams’ Application of Skills, Resources, and Abilities Within Their Context) by Month of Support

*Note*. CPC = core practice component.

**Figure 10:**
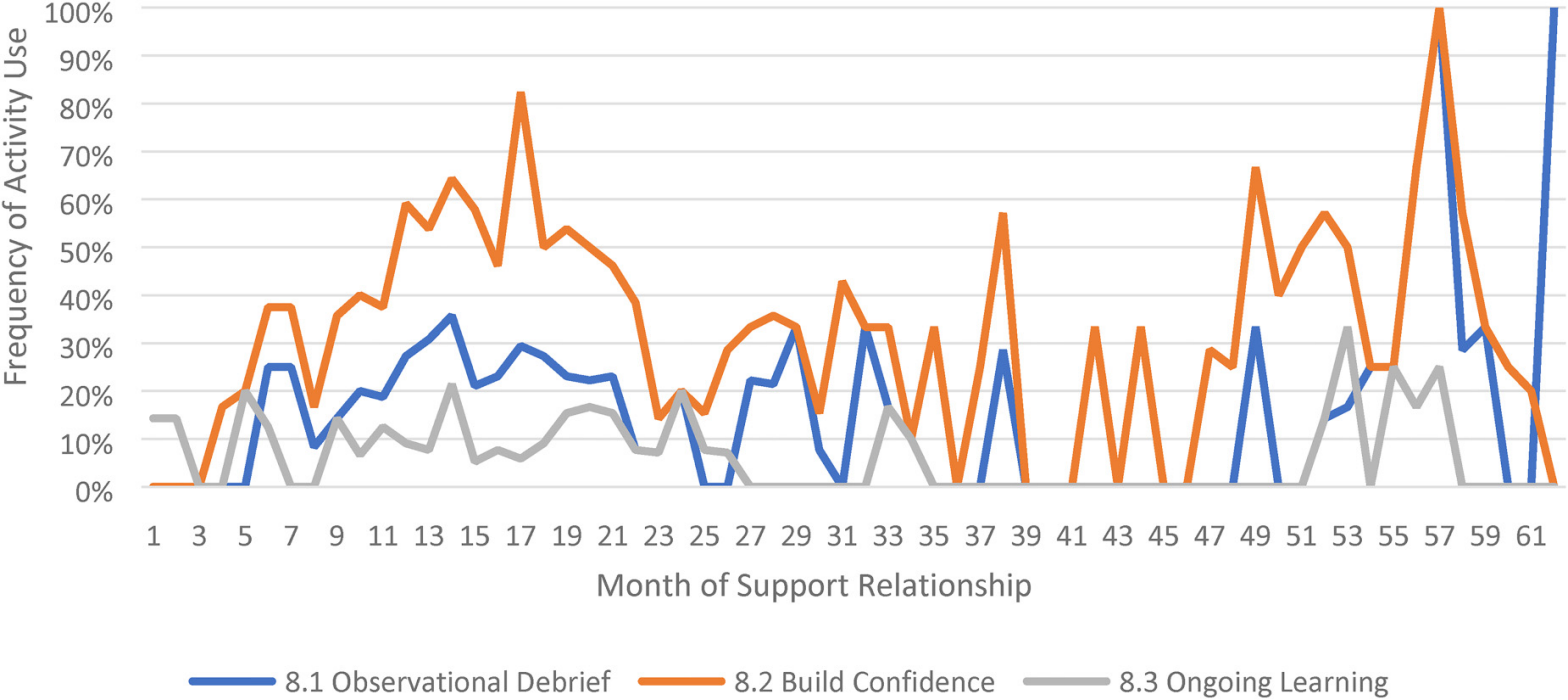
Use of Essential Activities Within CPC 8 (Provide Supportive Behavioral Coaching to Leaders and Teams) by Month of Support

*Note*. CPC = core practice component.

**Figure 11:**
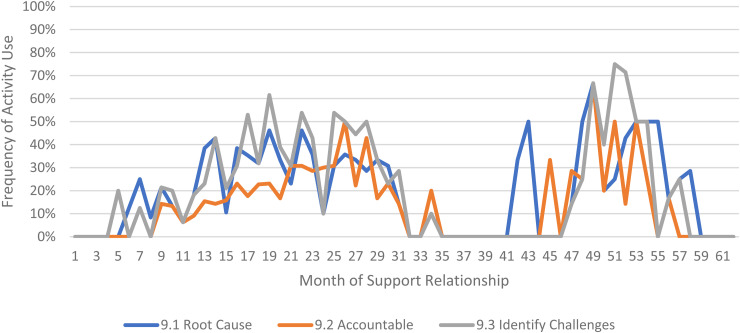
Use of Essential Activities Within CPC 9 (Facilitate Collective Learning and Adaptive Problem Solving) by Month of Support

*Note*. CPC = core practice component.

**Figure 12:**
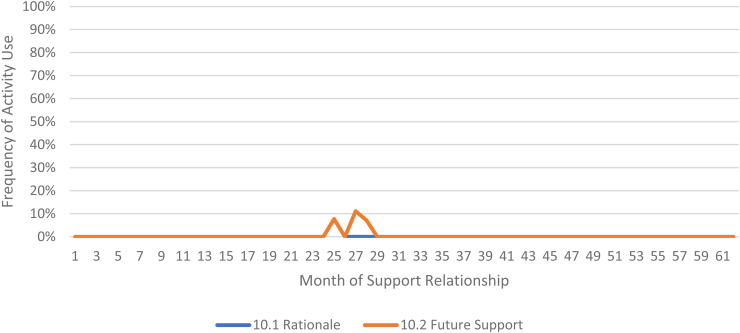
Use of Essential Activities Within CPC 10 (Transition Out of Intensive Implementation Support) by Month of Support

*Note*. CPC = core practice component.

We limited our examination of practice activities from July 2019 through December 2021, as noted above. During this time, 507 ICTP support events were tracked across all 13 regions. The proportional occurrence of practice activities within each CPC is reported in Table 5. The use of essential activities within each CPC are visualized in run charts in Figures 3–12. In these run charts, frequency of activity use each month was divided by the number of support events logged each month to normalize data where months have differing numbers of support events (Min. = 1, Max. = 22) and/or a different number of regions contributing data to each month (*n* = 1 to *n* = 11). See Appendix D for data used in these calculations.

**Table 5:** Frequency of Practice Activity Use within Core Practice Components

CPC	Frequency of each practice activity when CPC employed						
CPC 1	1.1^a^	1.2^a^	1.3^a^	1.4	1.5	1.6	1.7
	24%	86%	87%	88%	10%	14%	1%
CPC 2	2.1^a^	2.2^a^	2.3^a^	2.4^a^	2.5^a^		
	82%	66%	86%	66%	31%		
CPC 3	3.1^a^	3.2^a,b^	3.3i^a,b^	3.3fu^a,b^	3.4	3.5	
	58%	38%	8%	50%	15%	3%	
CPC 4	4.1^a^	4.2^a^	4.3^a^	4.4	4.5		
	62%	48%	47%	55%	42%		
CPC 5	5.1^a^	5.2^a^	5.3	5.4	5.5		
	33%	66%	69%	28%	20%		
CPC 6	6.1^a^	6.2^a^	6.3	6.4	6.5		
	63%	67%	39%	20%	26%		
CPC 7	7.1^a^	7.2^a^	7.3				
	85%	22%	44%				
CPC 8	8.1^a^	8.2^a^	8.3^a^	8.4	8.5		
	43%	92%	70%	53%	19%		
CPC 9	9.1^a^	9.2^a^	9.3^a^	9.4	9.5		
	66%	48%	73%	52%	40%		
CPC 10	10.1^a^	10.2^a^	10.3				
	33%	100%	33%				

*Note*. Analyses related to essential activities and practice enhancers are limited to tracking forms that were completed from July 2019 onward. CPC = core practice component.

^a^ Essential activities.

^b^ A confusion was identified among ICTP Regional Support Teams in coding activities 3.2 and 3.3. This confusion suggested that activity 3.2 was likely more used than data indicate and that activity 3.3fu was much less used than data indicate.

Examining Table 5, several observations are noteworthy. First, practice activities identified as essential activities are generally characterized by higher frequencies of use than practice activities that are not identified as essential. That said, a handful of practice enhancers, specifically 1.4 (check-ins), 4.4 (prioritization of goals), 4.5 (determine early wins), 5.3 (just-in-time learning), 7.3 (anticipatory guidance), 8.4 (normalize thoughts/feelings related to application), 9.4 (facilitate documentation of learning/problem solving), and 9.5 (facilitate engagement of key partners), might be worthy of reconsideration as essential activities based on ISPs’ high rates of use and ISP feedback otherwise. Essential activities with lower rates of use, specifically 3.3 (records reviews), 5.1 (set/monitor learning objectives), 7.2 (ensure experiential learning), and 10.1 (establish shared rationale), may need further clarification or support among ISPs for intended use, or might be worthy of reconsideration as practice enhancers.

Examining Figures 3–12, additional noteworthy observations emerge within at least three CPCs. First, essential activities 2.1 (self-efficacy) and 2.3 (personal agency) are used more frequently and earlier than other essential activities in this CPC (see Figure 4). Activities 2.2 (self-management tools) and 2.4 (problem solving) initiate later but are increasingly used as the relationship progresses. Although activity 2.5 (self-sufficiency) is used least often, use of this activity increases after the 1st year of support. Second, activity 5.3 (just-in-time learning) was used more frequently than 5.2 (proactively planned adult learning), especially in the first 18 months of the support relationship (see Figure 7). However, use of activity 5.3 decreases after the 1st year of support while 5.2 maintains a relatively stable frequency of use across the support relationship. Third, activity 6.1 (shared action plans) appears to follow a cyclic pattern, initiating after 4 months and being increasingly used before decreasing every 15–18 months over the course of the relationship (see Figure 8). Activity 6.2 (organizational change) follows a similar cyclic pattern, though tends to start earlier in the support relationship.

## Discussion

The goals of this study were to present descriptive analyses of EIS as operationalized and utilized within a project supporting regional partners scaling an evidence-based system of parenting and family supports in two U.S. states. The results provide one example of how EIS CPCs and practice activities may unfold over long-term support engagements. The results show promise for the ability to systematically and prospectively track ISP practice activities associated with potential mechanisms of change that may be linked to practice outcomes.

Several findings regarding overall support dose warrant discussion. First, support tapered over time, starting higher, leveling off at 2–3 h of support per month at the end of Year 1, and decreasing on average again in Years 2 and 3. This pattern might suggest that regions were developing greater implementation capacity and self-regulated performance over time, leading to less dependency on EIS and thus lower dose of support. The pattern might also suggest diminishing energy or effort among participants to engage in support over time. However, given that support dose tends to increase among the longest engaged support participants after Month 44 of support, energy and effort may be more dynamic over time or moderated by variables such as initial interest and ability to engage in support.

Second, there were differences in dosage across the EIS initiation year. Regions that initiated support before 2019 had a higher average monthly dose of EIS compared to regions that began after 2019. Given that regions were onboarded to the support process using a request for interest and assessment process related to engagement in intensive support, one interpretation is that regions that were more interested and able to engage in intensive support did so. Given the developmental nature of the practice model and practice teams over the course of this study, ICTP regional support teams also may have become more experienced and efficient providing streamlined iterations of EIS to regions with later start dates.

Third, South Carolina regions received lower average doses of support than regions in North Carolina. As the first two South Carolina regions were just starting their Triple P scale-up efforts, they were more regularly engaged by other intermediary supports and their state Triple P leadership partners, which may have reduced bandwidth for ICTP regional support activities. Relatedly, ICTP teams were more heavily focused on general implementation support than program-specific implementation support, which was primarily being provided by Triple P's purveyor organization. Therefore, results might suggest that the former is a less critical focus in early stages of implementation. Finally, the third South Carolina region had a known challenge with bandwidth to engage in intensive support due to having only one regional Triple P coordinator instead of a full regional implementation team.

The dosage of individual CPCs across the more than 5-year period provides helpful information to characterize key features of the support process, including when certain support activities may be more likely to take place. For example, in this study, EIS generally began with a high dose of support largely related to “foundational” and “co-design” CPCs (Aldridge et al., 2023). This may suggest key features and timelines for early phases of co-created support processes (Yazejian et al., 2019). That CPC 1 (collaborative relationships) was the most frequently used core component overall is consistent with other literature suggesting the key role of relationships in the support process (e.g., Albers et al., 2020; Chilenski et al., 2016; Katz & Wandersman, 2016). Support after 6 months was characterized by a more diverse composition of CPCs with certain CPCs ebbing or flowing at different times thereafter. Interestingly, CPC 8 (supportive behavioral coaching) was used earlier and at higher dosages than CPCs 7 (facilitate application) and 9 (collective learning and adaptive problem solving), suggesting it might have some independent utility in ISP efforts to support local leaders’ and teams’ effective implementation performance. CPC 10 (transition) was rarely used, but it was used in at least two regions that thereafter continued engagement in EIS, suggesting it might have interim utility in the support process around events such as transitioning support focus from one area of implementation performance to another. Together, these patterns suggest alignment with the dynamic and multi-path nature of EIS described by Aldridge et al., (2023) related to their conceptual model. Although the appearance of some sequential practice activities early in support may reflect similar early-stage concepts from stage-based models of implementation (e.g., Aarons et al., 2011), ICTP regional support teams more often engaged regional Triple P partners after they were several years into implementation efforts. The extent to which stages of implementation at the local level may influence or interact with EIS activities is an interesting area for future investigation.

The findings are also helpful in considering which practice activities may be essential activities, at least based on ISP rates of utilization. Furthermore, they suggest that individual practice activities even within CPCs may emerge at different times in the support engagement. For example, in our study, early ISP activities to reinforce self-regulation of effective implementation focused on personal agency (e.g., *Our team is responsible* for using effective implementation strategies) and self-efficacy (e.g., *Our team can* use effective implementation strategies), while later activities focused on self-management tools (e.g., Our team has *the resources we need* for effective implementation), problem solving (e.g., Our team can *identify and respond effectively to implementation challenges*), and, finally, self-sufficiency (e.g., Our team can manage implementation effectively *without dependence on EIS*; Roppolo et al., 2019). Just-in-time learning activities regarding implementation strategies were used more frequently than proactively planned learning activities, especially early in the support relationship. Such findings may provide insight into the relevance of, or support participants’ openness to, different support activities at different times. That essential activities within CPC 6 (develop implementation capacity) follow 15- to 18-month cyclical patterns may imply temporal characteristics related to organizational improvement or change processes. Overall, these findings may also suggest characteristics about ISP decision-making regarding practice strategies at different timepoints within the support engagement. They also align with the blended use of practice activities as discussed earlier and in Appendix B.

### A Practical Report From the Field

Although findings from the current study lend themselves to several directions regarding EIS, they also need to be appropriately recognized within the context of this practical report from the field. This study utilized one conceptual model of EIS across two statewide practice settings similarly characterized by regional, cross-sector scale-up of an evidence-based system of parenting and family supports. While the longevity of data collection provides several benefits, there were no comparison groups, randomization, or other controls. Moreover, results related to dosage are inclusive of data reflecting three iterations of our operationalized practice model. While we were able to reconcile practice activities for the most part, we cannot exclude the possibility of inconsistencies, particularly as ICTP regional support teams were learning the model as it was iteratively developed and applied. ICTP ISPs were also not immune to typical self-report biases, despite the supports provided around the use and tracking of the practice model.

Several contextual factors across the two statewide practice settings also may be important to recognize. As with many community initiatives based in child and family-serving organizations, staff turnover was constant and may have influenced the trajectory of support. During periods of staff transitions, ICTP regional support teams reported that EIS shifted to onboarding and revisiting partnership agreements, which may have influenced the use of certain CPCs and the overall timeline of support. ISPs from the Impact Center at FPG were not the only members of ICTP regional support teams during the 5 years of data collected for this study. The backgrounds and experiences of ISPs from partner organizations may have influenced support trajectories during the times in which they were engaged. Likewise, the delays providing foundational training in our practice model to ISPs from partner organizations (see Appendix C) may have impacted their early delivery of EIS. Relatedly, other intermediary and purveyor supports provided by other support system partners to regional Triple P partners in both states may have shaped EIS trajectories through parallel influences or reducing bandwidth to engage in EIS.

Like all field studies occurring within the past 2 years, the global COVID-19 pandemic was emerging or ongoing during part of this study. Although a report of how the pandemic influenced trajectories of EIS across regions participating in support is beyond the scope of the current study, it should be expected that some meaningful level of influence occurred, particularly given that many regions were led by a county or regional health department. Support during the pandemic transitioned entirely to virtual support and local health departments had intensive roles combating the pandemic that likely limited the ways and amount of time spent engaging in EIS for Triple P scale-up. Additionally complicating the pandemic era for North Carolina Triple P regions was a 1-year requirement from state Triple P leadership to engage in community-driven, data-based, 5-year strategic planning efforts. The support needs for this type of planning activity are unique in any context but required additional attention to providing practical and emotional support and facilitating collective learning and problem solving in the higher-stress, virtual work context of the pandemic.

### Future Directions

This study generates multiple directions for future EIS research. Foremost, additional descriptive studies of these or similar CPCs and practice activities in the field may provide new insights about their use and trajectories. Studies about the feasibility of participating in this type of highly tailored, long-term EIS may offer critical perspective from support participants. Qualitative studies of ISP experiences providing EIS according to this or other practice models may be helpful to uncover contextual decision-making patterns or choice points in the provision of EIS. Finally, the critical question remains whether use of this practice model as intended influences practice outcomes. While the ability to statistically test such associations is complicated by the small sample within this field trial (*n* = 13), some representation of possible associations may be important for continued momentum and to guide revisions. Although the question is beyond the scope of the current study, we are presently exploring methods to examine such associations with our available data sets.

As there are few studies reporting theoretically informed details about the provision of EIS, we have provided a level of detail that we hope proves useful to both researchers and ISPs. However, to support concision in future empirical reports, particularly wherein practice outcomes are simultaneously examined, we recommend authors focus on details related to the:

- models of EIS informing study activities, including underlying theory, CPCs and their operationalization, and theories of change for influencing intended practice outcomes;
- settings in and purposes for which EIS was provided;
- ISPs engaged in EIS; and
- participants in EIS and other intended beneficiaries.

Future directions for practice may include additional revisions to how CPCs have been operationalized. Increasing the clarity and usability of this practice model enables its systematic and intentional use by ISPs. Findings from this study may also help improve transparency in expectations regarding time investments and timelines for support participants and in generating shared values and norms between ISPs and support participants. For example, understanding the higher dose of support earlier in the relationship may help participants plan their time and effort accordingly. Likewise, understanding the variety of practice activities that might be expected in the broader timeline of support might improve participants’ receptivity to longer-term EIS and reinforce the value of early foundational and co-design activities. This may also help mitigate participant concerns about the narrow foci of early support during which they may experience pressures to use support to accelerate their implementation efforts.

## Appendix A

### Operationalizing the Core Practice Components: Description of Process and Iterative Improvements

To ensure that core practice components (CPCs) were both usable and trackable by implementation support practitioners (ISPs) providing external implementation support (EIS) to regional partners through the ICTP projects (hereafter referred to as *ICTP regional support teams*), a practice model leadership team within The Impact Center at FPG first met over several working sessions in spring and early summer 2017 to detail practice activities that could be used to describe and report on each CPC. The need to be more detailed about the elements of broad implementation strategies has been recognized in the field (Albers et al., 2021). For example, to describe CPC 1 (build collaborative relationships), three activities were initially outlined, including “share food (lunch, dinner, drinks) off-site”; “personal check-ins from the ISP”; and “support emotional, practical buy-in from the implementation team and leadership.” The resulting list of 59 practice activities across the 10 CPCs was unchanged from August through December 2017.

In late 2017, feedback from ICTP regional support teams indicated the need to revise several activities to enhance clarity and utility. To respond to this feedback, the practice model team met again over several working sessions. The only CPC with major revisions to its operationalization was CPC 1 (build collaborative relationships). Seven activities were added, increasing the number from three to 10, and wording edits were made to two of the original three activities. Examples of activities added included, “clarify partnership roles and expectations [initial visit]”; “collaboratively develop group agreements (values, behavioral norms) [initial visit]”; and “discuss hopes, concerns, critical context needs [initial visit].” An example of a wording edit was to clarify that “personal check-ins from the ISP” might be about either “work or life more broadly.” Moderate changes, reflecting the addition of one activity and minor wording edits to a small number of original activities, were made to each of CPCs 5, 6, and 7. Minimal changes, including only minor wording edits to a small number of activities, were made to CPCs 3 and 4. For CPCs 9 and 10, several original activities were identified as examples of another activity and thus collapsed together, though one new activity was also added for CPC 9. No changes were made to CPCs 2 or 8. The revised set of 60 practice activities was unchanged from January 2018 through June 2019.

In the first half of 2019, additional feedback from ICTP regional support teams indicated a need for further revision. Regional support teams described that practice activities were not often unfolding in the occasionally sequential ways they had been outlined within some CPCs. For example, some of the activities that had been designated for a particular support visit or as a prelude to other support activities were instead happening dynamically. Additionally, some of the support resources or tasks described were too specific to reasonably describe how activities were generally used in practice.

To respond to this second round of feedback, the practice model team reconvened to make a third pass on the practice activities. Several substantial changes were made. First, practice activities were centered closer to generalizable practice behaviors that might be used by ISPs on any support engagement or in any practice setting. Although many of the practice activities from previous iterations were already generalizable, several included wording, context cues, or support resources that were specifically developed within the ICTP projects. Relatedly, suggestions that some practice activities may happen within certain support visits or time periods were removed, allowing for a more fluid and dynamic use of practice activities across all CPCs. Finally, the practice model team reviewed and classified practice activities as either essential or nonessential, but still of high value. *Essential activities* were hypothesized to directly contribute to achievement of the intended near-term outcomes of each CPC (see Figure 1). Practice activities classified as nonessential, but still of high value and generally expected, were labeled *practice enhancers*. These practice activities were hypothesized to accelerate or otherwise enhance the realization of intended near-term practice outcomes, even if outcomes may be sufficiently achievable in their absence. Activities that did not attain consensus for being in either category were removed from the operationalization of the CPCs. Decisions were made based on the considerable practice experiences and advanced behavioral training across the practice model team and the feedback and experiences of ICTP regional support teams since applying the practice activities in the summer of 2017. The resulting 48 practice activities (28 essential activities and 20 practice enhancers) are reported in Table 1 and have remained unchanged since July 2019.

## Appendix B

### Intended Use of CPCs and Practice Activities: Additional Details and Examples

Activities related to the two foundational CPCs described by Aldridge et al., (2023), *Build Collaborative Relationships* and *Reinforce Leaders’ and Teams’ Self-Regulation of Effective Implementation Performance*, might be expected in most support interactions. For example, even in a support interaction well into the engagement, attention to building or maintaining a collaborative relationship with support participants using one or more related practice activities may be wise, if not necessary. Likewise, using one or more practice activities in early support interactions to reinforce self-regulation of existing elements of effective implementation performance may boost participants’ confidence that they can apply implementation best practices and reach their intended goals without long-term dependence on ISPs. This may also grow participants’ appetites to learn about and apply additional implementation strategies, particularly in areas where performance may be more developmental. As visualized in Figure 1, practice activities from these two CPCs are essential to setting a hospitable environment for the entire support engagement and to generalizing and sustaining the change process even after intensive support has ended.

Beyond these two foundational CPCs, the proactive objectives and responsive demands within any single support interaction might determine which additional practice activities are employed by the ISP. For example, if a primary objective of a support interaction is to further develop a team of support participants’ knowledge, skills, and abilities for effective implementation following their initial application of a newly learned implementation strategy or best practice, the ISP might:

- debrief with team members their learning from the application exercise (practice activity 8.1),
- provide specific behavioral praise for team actions that were aligned with the implementation strategy of interest (practice activity 8.2),
- engage in additional learning activities for team skill refinement in areas of performance needing additional improvement (practice activity 8.3),
- reinforce the team's own abilities to identify and address any challenges that may have emerged on the periphery of the initial application (practice activity 2.4),
- facilitate the team's identification of a next opportunity to apply the implementation strategy in a new or more refined way or to respond to peripheral challenges that have emerged (practice activity 7.1), and
- support the team's emotional and practical readiness for the next identified action step (practice activity 1.3).

In combining these practice activities, not only has the ISP made efforts to further develop team members’ knowledge, skills, and abilities for effective implementation (through CPC 8), but also to reinforce the team's self-regulation of implementation practices (through CPC 2), facilitate the team's ongoing learning and skill refinement (through CPC 7), and strengthen the working alliance (through CPC 1).

Alternatively, it may not be unusual in an example like this for the ISP to:

- themself facilitate the team's problem-solving process about any challenges that have emerged (CPC 9) rather than to reinforce the team's own self-regulation of the problem-solving process (CPC 2),
- assess organizational or system factors about any challenges that have emerged (CPC 3), and/or
- introduce new implementation strategies (CPC 5) that might respond to the emergent challenges.

There are many factors that might influence these various choice points among ISPs, including momentary priorities and preferences from support participants, the ISPs overarching conceptualization of the implementation site's performance, the time available in the support interaction, and other priorities on the near-term agenda of the support engagement.

## Appendix C

### ICTP Regional Support: ISP Training and Coaching, Support Participants, and Details About Statewide Contexts and Support Partnerships

#### ICTP ISP Training And Coaching

All ICTP ISPs were provided foundational training on the broad elements of the practice model within their first several months on the ICTP projects. This training was provided by senior members of the ICTP project team, including the first author. Training typically consisted of 12–17 sessions, often 2–3 h in length, integrating presentation and active learning exercises. In addition to participating in monthly project management meetings, at least monthly (typically twice monthly) opportunities for practice coaching were provided to ICTP ISPs. Coaching activities have typically included case consultation, data review, and additional learning opportunities about practice resources and approaches. When systematic coaching supports were less available for a period (e.g., due to transition of the team member leading coaching activities), senior team members, including the first author, remained available for practice coaching as needed or requested. All ICTP ISPs were also provided regular opportunities to participate in in-service professional development activities through professional conferences and webinars or as otherwise led by members of the Impact Center at FPG and its collaborators. Topics typically covered novel or less familiar approaches and resources relevant to implementation practice from the broader field.

#### Participants in ICTP Regional Support

Within each region, a community Triple P implementation team, consisting primarily of two-to-three funded regional Triple P coordinators, ensures regional Triple P scale-up and support activities. These individuals are housed within a regional Triple P backbone organization. The executive leader within the backbone organization (typically the organizational director) is accountable for regional Triple P activities and either stays directly connected with the community implementation team or delegates supervision and support to an upper manager. Regions may also recruit and organize broader community Triple P leadership across an array of community provider organizations and partners focused on providing or advocating for child and family supports. When well organized, these broader community Triple P leadership teams foster shared ownership of Triple P scale-up efforts and nurture broader community engagement, equity, and inclusion in regional Triple P system designs. Local service providers are typically selected by community Triple P implementation team members and leaders to implement programmatic variants of the Triple P system, started by sending one or more practitioners to Triple P program training provided by Triple P America.

Although ICTP ISPs provide support to the entire regional partnership involved in Triple P scale-up efforts, the primary locus of their influence is through support provided directly to community Triple P implementation team members and leaders. This support approach intends to position these individuals to be active, sustainable agents in creating the changes their communities need to support and sustain Triple P scale-up. This also promotes self-regulation and minimizes long-term dependence on ICTP regional support teams for *intensive* supports (Aldridge et al., 2023). Although the practice model is intended to be applied consistently across regional support partnerships, contextual differences typically present the need for tailored use of practice principles, components, and activities.

#### North Carolina Context and Support Partnerships

In North Carolina, the Division of Public Health started state-funded Triple P system scale-up in 2012 with five counties and continued to expand Triple P scale-up by the addition of cohorts of counties through March 2013. In 2016, the then 37 scale-up counties were reorganized into “clusters” when several counties originally supported through time-limited federal funds were transferred to state funds. In mid-2018, the Division of Public Health and Division of Social Services, which had also started funding Triple P scale-up in 2017, adopted a statewide model consisting of 10 regions to provide coverage for Triple P implementation and scale-up. Each region is supported by a regional backbone organization that is responsible for an array of community backbone functions, such as mobilizing available state funds, convening community partners, facilitating dialogue, facilitating community-driven strategic planning, managing data collection, coordinating outreach, and supporting Triple P provider organizations and their practitioners (North Carolina Triple P Partnership for Strategy and Governance, 2020). In nine regions, the backbone organization is a county or regional health department. In the lone region funded by a private endowment, the backbone organization is the local school district. Regions range from a single county to a 17-county catchment area. Not every county in each multi-county region is scaling the full Triple P system. Regardless, the regional backbone organization acts as a support partner for counties with limited Triple P implementation (e.g., a small number of provider organizations implementing Triple P variants) or exploring future Triple P system scale-up. Currently, there are approximately 38 scale-up counties and 62 non-scaling counties (a mix of counties with some Triple P implementation, some Triple P exploration, and no Triple P activity) across the state.

From July 2019 through June 2020, ICTP ISPs partnered with parenting program specialists from a second statewide intermediary organization to provide tailored EIS. This ensured adequate capacity for intensive support as new regions were onboarded to the support process. This second intermediary organization was already supporting other Triple P intermediary functions in the state (e.g., partnership engagement and communication, policy and finance support, and elements of workforce development; Mettrick et al., 2015) and had a history of providing practitioner coaching and brief, responsive elements of implementation support for other parenting programs in the state. Parenting program specialists from the second intermediary organization were provided training in the broad elements of the practice model and tracking strategies being used by the ICTP project team in October 2019. This training was provided by senior members of the ICTP project team, including the first author. Efforts to provide this training during a June 2019 workshop were unsuccessful due to scheduling conflicts. To bridge the gap from July 2019 until October 2019, ICTP ISPs paired on regional support teams with a parenting program specialist provided individualized consultation in the use of the practice model and tracking strategies. Ongoing coaching support for the parenting program specialists following the October 2019 workshop was provided by their in-house supervisors, two of whom attended the October 2019 workshop. In July 2020, the ICTP project team resumed sole responsibility for providing broad-based EIS to Triple P regions across the state.

#### South Carolina Context and Support Partnerships

In 2018 in South Carolina, a statewide intermediary organization with a history of supporting prevention program and policy implementation in the state started funding Triple P system scale-up in two counties with support from a regional endowment. Similar to the North Carolina Triple P model, community backbone organizations were selected and funded to support the scale-up process. In South Carolina, this selection process included a request for interest and assessment process inclusive of elements related to both Triple P system scale-up and participation in an intensive support process. The two selected backbone organizations in South Carolina were a local community health organization and a local community early childhood organization. The first county started Triple P scale-up in October 2018, and the second county in January 2019. Tailored EIS was provided to both counties from the start. In May 2019, a third county that had been previously scaling the Triple P system was added to the support process. The backbone organization in the third county was a child and family-serving organization funded separately by a local foundation.

Similar to the model used in North Carolina, community capacity coaches (Strompolis et al., 2020) from the South Carolina intermediary organization partnered with ICTP ISPs to provide tailored EIS to South Carolina Triple P counties. Community capacity coaches were provided training in the broad elements of the practice model by senior members of the ICTP project team, including the first author, in June 2019. Interests to provide this training earlier were delayed by competing priorities such as hiring and onboard processes across both the South Carolina intermediary organization and the ICTP project team, which was filling open ISP positions during this time. To bridge the gap from fall 2018 until June 2019, ICTP ISPs paired on regional support teams with a community capacity coach provided individualized consultation in the use of the practice model. Ongoing coaching support for the community capacity coaches following the June 2019 workshop was shared across ICTP ISPs, ICTP senior team members, and by their in-house supervisor, who attended the June 2019 workshop as well.

## Appendix D

### Summary of CPC Use by Month of Support, July 2019 Through December 2021

Details of the 507 support events tracked from July 2019 through December 2021, including frequency of CPC use and the number of regions contributing data to the analysis for each support month.[Table table6-26334895231154285]

**Table table6-26334895231154285:**

Support month	# Regions	# Events	CPC 1 used	CPC 2 used	CPC 3 used	CPC 4 used	CPC 5 used	CPC 6 used	CPC 7 used	CPC 8 used	CPC 9 used	CPC 10 used
1	5	7	100%	0%	43%	0%	0%	0%	0%	14%	0%	0%
2	5	7	100%	43%	29%	43%	29%	0%	0%	14%	0%	0%
3	5	5	100%	60%	60%	40%	40%	20%	0%	0%	20%	0%
4	5	6	83%	50%	50%	33%	33%	33%	0%	17%	0%	0%
5	5	5	100%	80%	20%	60%	60%	60%	20%	20%	20%	0%
6	7	8	75%	38%	13%	38%	13%	63%	0%	50%	25%	0%
7	7	8	100%	63%	38%	75%	38%	63%	25%	38%	25%	0%
8	8	12	100%	67%	25%	50%	33%	33%	8%	17%	17%	0%
9	10	14	93%	64%	7%	14%	43%	43%	21%	36%	43%	0%
10	10	15	87%	53%	47%	40%	27%	40%	33%	40%	27%	0%
11	11	16	88%	50%	25%	13%	63%	31%	13%	44%	25%	0%
12	11	22	95%	50%	18%	14%	41%	59%	50%	59%	32%	0%
13	11	13	92%	77%	23%	23%	38%	31%	38%	54%	46%	0%
14	11	14	79%	64%	21%	21%	43%	50%	29%	64%	57%	0%
15	11	19	84%	68%	32%	26%	37%	37%	42%	58%	21%	0%
16	11	13	100%	77%	31%	38%	23%	15%	31%	46%	38%	0%
17	11	17	82%	71%	35%	18%	29%	41%	47%	82%	53%	0%
18	11	22	77%	50%	14%	32%	18%	18%	27%	50%	41%	0%
19	11	13	92%	62%	23%	31%	23%	15%	38%	62%	69%	0%
20	11	18	72%	56%	11%	22%	39%	44%	33%	56%	39%	0%
21	10	13	92%	77%	38%	15%	31%	38%	38%	54%	38%	0%
22	10	13	62%	77%	31%	31%	23%	38%	23%	38%	69%	0%
23	9	14	100%	86%	36%	29%	7%	50%	14%	29%	50%	0%
24	8	10	100%	80%	70%	40%	10%	40%	30%	40%	30%	0%
25	8	13	100%	69%	62%	38%	31%	38%	0%	38%	69%	8%
26	7	12	79%	64%	71%	29%	43%	57%	21%	29%	50%	0%
27	7	9	89%	89%	33%	22%	0%	100%	11%	33%	44%	11%
28	7	14	93%	79%	43%	14%	29%	43%	21%	36%	50%	7%
29	7	6	100%	83%	67%	0%	17%	67%	0%	33%	50%	0%
30	7	13	100%	62%	38%	31%	15%	8%	8%	15%	38%	0%
31	5	6	86%	71%	43%	14%	29%	71%	0%	43%	29%	0%
32	5	3	100%	100%	33%	0%	67%	33%	33%	33%	0%	0%
33	6	6	100%	67%	67%	33%	17%	33%	33%	33%	0%	0%
34	6	10	100%	50%	70%	20%	30%	50%	10%	20%	30%	0%
35	6	6	100%	33%	67%	17%	17%	83%	17%	33%	0%	0%
36	4	5	67%	33%	50%	50%	50%	50%	17%	0%	0%	0%
37	4	4	100%	50%	75%	25%	50%	50%	0%	25%	0%	0%
38	3	5	57%	29%	43%	0%	29%	14%	43%	57%	14%	0%
39	2	2	67%	67%	0%	0%	0%	0%	33%	0%	0%	0%
40	2	2	100%	100%	0%	0%	0%	0%	0%	0%	50%	0%
41	2	1	0%	100%	100%	0%	0%	0%	0%	0%	0%	0%
42	2	3	100%	100%	33%	33%	0%	67%	33%	33%	33%	0%
43	2	2	100%	50%	0%	50%	0%	0%	0%	0%	50%	0%
44	2	3	100%	100%	0%	0%	33%	67%	0%	33%	0%	0%
45	2	3	100%	100%	67%	67%	33%	0%	0%	0%	33%	0%
46	2	3	67%	67%	0%	33%	33%	67%	0%	0%	0%	0%
47	2	7	86%	43%	29%	43%	29%	43%	0%	29%	29%	0%
48	2	4	100%	100%	0%	25%	50%	50%	25%	25%	50%	0%
49	2	3	100%	100%	33%	33%	33%	67%	0%	67%	67%	0%
50	2	5	60%	60%	0%	0%	0%	60%	20%	40%	40%	0%
51	2	4	100%	100%	50%	25%	25%	50%	25%	50%	75%	0%
52	2	7	86%	86%	0%	29%	29%	43%	29%	71%	71%	0%
53	2	6	83%	83%	17%	0%	17%	50%	0%	50%	50%	0%
54	2	4	100%	75%	25%	0%	25%	25%	0%	25%	50%	0%
55	2	4	100%	75%	50%	0%	25%	75%	25%	25%	50%	0%
56	2	6	100%	67%	83%	17%	33%	67%	33%	67%	33%	0%
57	2	4	100%	25%	75%	0%	50%	75%	50%	100%	25%	0%
58	2	7	100%	57%	100%	14%	57%	57%	14%	57%	29%	0%
59	2	3	100%	67%	100%	0%	33%	33%	100%	33%	0%	0%
60	1	2	50%	50%	50%	50%	50%	25%	50%	25%	0%	0%
61	1	5	100%	60%	100%	0%	40%	20%	100%	20%	0%	0%
62	1	1	100%	100%	100%	0%	100%	100%	100%	100%	0%	0%

*Note*. CPC = core practice component.
